# Supplementary material for: Patients’ attitudes toward artificial intelligence (AI) in cancer care: A scoping review protocol
Source: PLoS One. 2025 Jan 14;20(1):e0317276. doi: 10.1371/journal.pone.0317276 (PMC11731723; doi:10.1371/journal.pone.0317276)
Supplement: Attachment — Submitted filename: Response to Reviewers.docx [file pone.0317276.s003.docx]

Rebuttal Letter - Patients’ attitudes towards artificial intelligence (AI) in cancer care: a scoping review protocol

Dear Reviewers and Editor,

Thank you for your consideration and valuable feedback for our manuscript.

We have addressed each reviewer comment below, and included the relevant changes to the text.

In particular, we thank Reviewer #1 for their suggestions around adding further methodological details to increase the rigor of this work. We have now added multiple extra details to stages 3, 4, and 6 of the methodology to ensure the methods are well described and replicable.

With thanks,

Daniel Hilbers, Navid Nekain, Alan T. Bates, and John-Jose Nunez

**Reviewer #1:**

1. **Line 72-73: Refers to specific authors without providing a citation number, which would allow the reader to draw a link between the author and a presumably prior mentioned studies.**

Thank you for your suggestion to include a citation number. We have provided a citation in the appropriate line.

2. **Line 75-77: There is likely to be a high degree of overlap between attitudes and perspectives vs emotions and feelings both within the respective combinations and between them. Inclined to disagree that exploration of emotions and feelings is a novel contribution in this context.**

Thank you for this comment. We agree that there is overlap between attitudes and perspectives vs emotions and feelings. We have removed the following sentence:

*“This review will further expand prior studies by not only including patients' attitudes and perspectives, but also explore the emotions and feelings of patients with cancer regarding AI in their medical care.”*

The remaining paragraph, lines 70-75, addresses the gap in the literature and highlights the novel contribution of this review. Specifically, it focuses on examining the attitudes of cancer patients towards the use of AI, rather than considering patients with all medical conditions or only those with breast cancer.

3. **Line 89: makes a sweeping comment about multiple areas which benefit from this specific type of review. It is not clear to me how a scoping review in particular can do what is claimed in line 89. requires more clarity.**

Thank you for your insight regarding the breadth and benefits of our scoping review. We agree that there can be further clarification to the benefits mentioned in line 89. We have revised the paragraph and have included the new text below:

*“The objective of this review is to identify knowledge gaps, map existing literature, and assess available evidence. To achieve this, we will conduct a scoping review, which is particularly suited for mapping the breadth of available research and identifying gaps where evidence is limited (18). Scoping reviews are also useful for clarifying definitions of key concepts in emerging fields, providing an overview of the research landscape, and highlighting areas where further study is needed (19,20). Additionally, they can facilitate stakeholder engagement by summarizing existing knowledge and guiding future research directions based on ongoing developments (20,21). We registered this protocol with the Open Science Framework (https://osf.io/8zph9).”*

The methodology for this scoping review adheres to widely accepted guidelines for scoping review methodology (18-21).

Methods section:

4. **Stage 5 lacks relevant details on undertaking the review such as whether inclusion and exclusion criteria will be determined using a framework such as PICO. What information will be extracted for quantitative vs qualitative vs mixed methods studies and whether a rating tool for quality of studies such as the MMAT will be used for this purpose.**

Thank you for this insightful comment. We have included the below sentence in stage 4 outlining the PCC framework used to determine the inclusion/exclusion criteria:

“Our inclusion and exclusion criteria were determined using the Population-Concept-Context (PCC) framework (23).”

We have opted to use the PCC framework over PICO framework as the PCC framework is preferred for a scoping review, since it aims to address questions outside of a specific intervention (19).

In table 3, we have outlined what information will be extracted from each of the selected studies.

In contrast to systematic reviews, scoping reviews aim to provide an overview of the existing evidence base without considering the quality of individual studies (20). Therefore, a formal assessment of methodological quality is typically not conducted in scoping reviews (20). As this review follows the scoping methodology, we will not include quality assessment of the studies.

5. **Stage 6: This step does not add methodological rigor to the research undertaken, as it is about dissemination of the findings, beyond completion of the work.**

We agree with this comment and removed the point of adding methodological rigor in stage 6. The text has been adjusted as follows:

“*We will consult with stakeholders and share the preliminary findings with patients with cancer, clinicians, and other relevant parties at an upcoming research summit workshop (21).*”

6. **Line 170: reference is made to end of life care, which is a stage of cancer. if this is part of the focus of the review, table 3 should also extract information regarding the stage of cancer, in addition to cancer type.**

Thank you for this comment. We have included cancer stage in table 3.

7. **Line 174-175: Are these the key distinctions between adult vs child and adolescent demographics? Consider aspects such as ability to provide informed consent here.**

We believe that the key demographic distinctions between adult and child patients with cancer that resulted in us excluding pediatric studies are the differences in cancer types and their familiarity with AI based technology. We have adjusted the text to include informed consent as a challenge with pediatric patients.

“*We believe that deferring cancer care for children and adolescents is justified due to the unique characteristics of this population, including the distinct types of cancers they face, expected differences in technological familiarity and comfort, as well as variations in autonomy and their capacity to provide informed consent (29,30).*”

8. **While there is likely to be value in the proposed work, the paper will benefit from consideration given to the above, and attention paid to grammar, typos, tense. Further detail is needed in the methods section to increase replicability of this protocol.**

Thank you for the above comments. We have thoroughly reviewed the paper and made the appropriate edits to grammar, spelling errors and tense. These edits can be seen in the revised manuscript.

Reviewer #2: This review presents opinion analysis of using AI in cancer care from the perspective of the patient. It is an important perspective to consider given the integration of AI in cancer care.

Following are some comments to improve:

**1. Consider using terms like privacy/security in search terms as these are major concerns for patients**

Thank you for this comment. We agree that a major concern for patients involves data privacy. However, the objective of our scoping review is to explore the thoughts, feelings, emotions, and perspectives of patients towards AI in their care. We have established our search criteria to focus on descriptive terms capturing patients' attitudes, rather than expected concerns of AI. We expect to identify papers that discuss patients' concerns regarding data privacy, but do not believe that we should include this in our search criteria, as it could narrow our results.

We have edited the following paragraph in our discussion to identify the expected theme of privacy:

*“Based on prior research, we anticipated that cancer patients would generally express optimism about the use of AI in their care. However, we also expected heightened concerns regarding its unsupervised use, potential privacy issues, and specific themes related to end-of-life care, particularly whether AI might be employed to discontinue curative-intent treatments.”*

**2. Explain how your study will change based on the consultation with stakeholders. What impact will it have? Clear explanation on this process will be important.**

Thank you reviewer, we have added additional details in stage 6 to how these consultations with be used:

*"Incorporating stakeholder consultation will enhance the methodological rigor of our research process in several key ways (19). First, stakeholder input is essential for ensuring that our findings address the most pressing concerns of patients, clinicians, and other relevant groups (27). By consulting stakeholders, we aim to capture important perspectives from patients that may otherwise be overlooked. This could lead to additional searches informed by stakeholder insights, identifying any missed studies or suggesting new directions for future research. Additionally, engaging stakeholders will help interpret and verify the accuracy of our results by incorporating their context and perspectives (28). Finally, stakeholder involvement will highlight opportunities for future research, as discussions are likely to uncover gaps and propose areas for further investigation (26). Specifically, we will seek stakeholder input on integrating our findings with future plans to implement AI tools in cancer care."*

**3. Consider including the stage in the cancer journey to the analysis (Context column in Table 3) as it can provide valuable insights, such as use of AI in diagnosis/treatment/post-care etc. Patients' opinions might differ based on for what purpose AI is used for.**

Thank you for this insightful comment. We have included the cancer stage in Table 3.
