# Supplementary material for: Patients’ attitudes toward artificial intelligence (AI) in cancer care: A scoping review protocol
Source: PLoS One. 2025 Jan 14;20(1):e0317276. doi: 10.1371/journal.pone.0317276 (PMC11731723; doi:10.1371/journal.pone.0317276)
Supplement: Attachment — Submitted filename: Response to reviewers Round 2.docx [file pone.0317276.s004.docx]

Rebuttal Letter - Patients’ attitudes towards artificial intelligence (AI) in cancer care: a scoping review protocol

Dear Reviewers and Editor,

Thank you for your consideration and valuable feedback for our manuscript.

We have addressed the reviewers’ comments below and included the relevant changes to the text.

With thanks,

Daniel Hilbers, Navid Nekain, Alan T. Bates, and John-Jose Nunez

Reviewer #1:

**The justification for focusing on cancer in this review is likely to benefit from outlining cancer statistics (i.e. mortality and mortality data, inequitable access to care, and the role of AI in addressing inequitable access for people who cannot easily attend specialised centres providing cancer care).**

Thank you for your feedback. We agree that outlining relevant cancer statistics such as mortality and access to care will greatly benefit the review’s justification for focusing on cancer. We have addressed this by adding text to the manuscript at the following lines in the introduction (Line 45):

“The application of AI in oncology has grown substantially in recent years (4,5). Cancer is the second leading cause of death worldwide, and disproportionately affects individuals with restricted healthcare access, especially in rural areas and low- and middle-income countries (6-8). AI has the potential to improve access to specialized care and address these health inequities, particularly by enhancing early detection and facilitating remote diagnostics (4).”

**Line 21: I don't agree that "chronicity and severity" is something that differentiates a cancer from "other medical conditions". There is a plethora of other medical conditions which can be equally chronic and severe.**

Thank you for bringing up this great point. We have addressed this by changing the text to:

“However, these views have not been systematically examined from the perspective of patients with cancer, whose opinions may differ given the distinct psychosocial toll of the disease.”

We have also opted to edit line 50 to change the word “unique” to “distinct” as we agree with your point mentioned here and believe that distinct better describes the psychosocial toll patients with cancer face.

“Patients with cancer can experience a distinct unique psychosocial toll, given the extended duration, severity, and chronic nature of their illness (10,11).”

**Line 44-45: Diagnosis is not limited to breast cancer. Would be good to expand on the other cancers for which AI is used as well.**

We agree that we should expand on other cancers for which AI is used. We have included the following text:

“Specifically, AI algorithms have demonstrated high accuracy in identifying malignant lung nodules, assisting radiologists in improving early detection and treatment decisions (8). In colorectal cancer, AI has been employed to detect polyps and other early-stage cancer indicators via colonoscopy and histopathology analysis (9). In mammography, AI enhances breast cancer detection by improving radiologists’ performance and accuracy particularly for early-stage cancers (10).”

**Line 143-144: Please reword to make it clear that patients' attitudes are being examined. Currently a grammar error in this sentence.**

Thank you for identifying this. We have adjusted the text to:

“Explore implications for future research: This scoping review will guide future research by examining the attitudes of patients with cancer attitudes toward the use of AI in their medical care (18,19,25).”

**Stage 6 : Stakeholder consultation as described is not a standard method of adding rigour to a systematic review. A review is conducted to scope the existing literature on a give topic and report on the findings from the literature, alone. Therefore, the statements in line 158 -160 in my opinion are not appropriate in this context, and in fact reduced the credibility of the review, as it is then influenced by external sources of information.**

**Stakeholder involvement, if at all, should be conducted prior to determining the scope of a review, the research questions and search terms. Not as the final stage.**

Thank you for identifying this and we value your input regarding the reduced credibility of involving stakeholder consultation in a scoping review. We have adjusted our text below to move away from emphasizing that stakeholder consultation adds methodological rigor. Specifically, we emphasize how this will improve the relevance and applicability of our research and perhaps assist in meeting one of our objectives of identifying areas for future research. We would like to emphasize that we are following a gold standard methodological approach for scoping reviews which suggests that stakeholder consultation is essential and should be conducted as stage 6 (21).

“Engaging stakeholders will enhance the relevance and applicability of our findings in several key ways (21). As this is a scoping review, our aim is to map existing literature, identify knowledge gaps, and outline opportunities for future research. Stakeholder input will ensure that the research addresses the most pressing concerns of patients, clinicians, and other relevant groups (28). By incorporating these perspectives, we aim to highlight potential gaps and additional areas of interest that may not be fully represented in the literature. Additionally, engaging stakeholders can assist in interpreting our findings by offering context that might be overlooked (29). This may highlight opportunities for future research, as discussions are likely to uncover gaps and propose areas for further investigation (30). Specifically, we will seek stakeholder input to help integrate our findings with future efforts to implement AI tools in cancer care.”

**While it is evident that effort to report more detail on the methodology has been undertaken by the authors, it is important the authors give due consideration to the concerns noted above, as these concerns have a direct impact on the methodology of this protocol as well as the resultant findings reported from this review.**

We are grateful for your time in reviewing our paper. You have brought forward some excellent points and we have done our best to adjust our paper accordingly.
